# Electronic Tongue as a Correlative Technique for Modeling Cattle Meat Quality and Classification of Breeds

**DOI:** 10.3390/foods10102283

**Published:** 2021-09-26

**Authors:** József Surányi, John-Lewis Zinia Zaukuu, László Friedrich, Zoltan Kovacs, Ferenc Horváth, Csaba Németh, Zoltán Kókai

**Affiliations:** 1 Department of Refrigeration and Livestocks’ Products Technology, Institute of Food Science and Technology, Hungarian University of Agriculture and Life Sciences, 43-45 Ménesi Street, H-1118 Budapest, Hungary; Suranyi.Jozsef@uni-mate.hu (J.S.); Friedrich.Laszlo.Ferenc@uni-mate.hu (L.F.); 2 Department of Measurements and Process Control, Institute of Food Science and Technology, Hungarian University of Agriculture and Life Sciences, 14-16 Somlói Street, H-1118 Budapest, Hungary; Kovacs.Zoltan.food@uni-mate.hu; 3 SPAR Hungary Kft., 0326/1 SPAR Street, H-2060 Bicske, Hungary; ferenc.horvath@spar.hu; 4 Capriovus Kft., 073/72 Dunasor Street, H-2317 Szigetcsép, Hungary; nemeth.csaba@capriovus.hu; 5 Department of Postharvest Science, Trade and Sensory Evaluation, Institute of Food Science and Technology, Hungarian University of Agriculture and Life Sciences, 35-43 Villányi Street, H-1118 Budapest, Hungary; kokai.zoltan@uni-mate.hu

**Keywords:** sensors, chemometrics, beef, quality control, e-tongue

## Abstract

Discrimination and species identification of meat has always been of paramount importance in the European meat market. This is often achieved using different conventional analytical methods but advanced sensor-based methods, such as the electronic tongue (e-tongue), are also gaining attention for rapid and reliable analysis. The aim of this study was to discriminate Angus, domestic buffalo, Hungarian Grey, Hungarian Spotted cattle, and Holstein beef meat samples from the chuck steak part of the animals, which mostly contained longissimus dorsi muscles, using e-tongue as a correlative technique with conventional methods for analysis of pH, color, texture, water activity, water-holding capacity, cooking yield, water binding activity, and descriptive sensory analysis. Analysis of variance (ANOVA) was used to determine significant differences between the measured quality traits of the five-meat species after analysis with conventional analytical methods. E-tongue data were visualized with principal component analysis (PCA) before classifying the five-meat species with linear discriminant analysis (LDA). Significant differences were observed among some of the investigated quality parameter. In most cases, Hungarian Grey was most different from the other species. Using e-tongue, separation patterns could be observed in the PCA that were confirmed with 100% recognition and 97.5% prediction of all the different meat species in LDA.

## 1. Introduction

Meat provenance and meat quality are important areas of focus for the European meat market, which provides approximately 30% of the available calories in the European Union (EU) through meat consumption [1]. Meat quality and labelling of its geographical origin or breed specification is regulated by the Regulation (Eu) No. 1169/2011 of the European Parliament and of the Council [2], since the genetic background belongs to the most important factors affecting meat quality.

Meat from dairy breeds has been generally considered of inferior eating quality, compared to British and European cattle breeds [3], such as the Hungarian Grey cattle (Szurkemarha), Holstein, Angus, Hungarian Spotted cattle (Magyar tarka), or even the domesticated buffalo (Bivaly). Consumers often prefer to know about the species of meat they consume for several reasons, ranging from cost, preferences, religion, nutritional requirements, or mere pleasure. The meat quality of cattle breeds has been measured and compared in numerous studies and comparisons are mostly concentrated on potential breed differences at a similar age, length of fattening period, and weight; additionally, it always important to use the same body part [4]. These mechanisms show joint effects of the production factors (gender, age, breed, feeding, etc.) on sensory attributes (texture, color, and flavor) [3], as well as the biological characteristics of the carcass (pH, water activity, dry matter content (DMC), water-holding capacity (WHC), juiciness, etc.) [5].

Currently, these quality markers are being monitored and determined through many different methods but the most common are spectrophotometric techniques (color) [6], pH differential methods (pH) [7], texture analyzers (for tenderness, juiciness, drip loss, etc.) [8], Soxhlet (crude fat), Kjeldahl analysis (crude protein) [9], and the filter-paper press method (WHC) [10]. Recent advances in technology have, however, led steady increase in the application of sensor-based technologies for food quality, primarily instruments composed of electrochemical sensors of which a major example is the electronic tongue (e-tongue).

The International Union of Pure and Applied Chemistry (IUPAC) defines the electronic tongue as “a multisensory system consisting of a number of selective sensors and uses advanced mathematical procedures for signal processing based on pattern recognition and/or multivariate data analysis” [11]. In comparison to the human tongue, the e-tongue has improvements in the sensitivity, selectivity, and multiplexing capacity of modern biosensors [12]. It is capable of providing rapid, real-time, accurate, and reliable data, regarding various samples under study and has gained fame in the pharmaceutical, cosmetics, environmental control, engineering (petroleum), agriculture, and food beverage industries. The e-tongue has reportedly been applied for detection of different forms of adulteration in foods, such as tomato paste [13], honey [14], paprika powder [15], etc. It has also been used for fingerprinting classification and prediction of quality parameters in different food types, such as olives [16], identification of flavor peptides in fish [17], and measurement of taste attributes in beverages [18].

One if its major advantages is that it is non-subjective and can be used for both targeted and non-targeted analysis, due to their partial/non-partial selectivity and sensitivity to different ionic compounds in solution [19]. In spite of these advantages, the e-tongue has scantily been applied for meat evaluations, primarily because the instrumental setup is most suitable for liquid products and is also susceptible to drift (conditions that affect sensor sensitivity). However, with proven drift corrections techniques [20] and emerging standardized methods of meat sample preparation, the instrument can be applied either independently [21] or as a correlative technique to determine meat fraud [22], predict meat origin [23], evaluate beef flavor [24] or baked bacon sensory attributes [25], and predict taste compounds in chicken [26]. With an increasing demand of major cattle breeds, such as the Hungarian Grey cattle, Holstein, Angus, Hungarian Spotted cattle, and domesticated buffalo, there have been unconfirmed rumors about differences in their sensorial quality, which could lead to issues of fraud. In addition, the e-tongue has never been applied to study meat quality differences in these cattle breeds. The potential to discriminate and differentiate these meat types at the industrial level using rapid methods, such as the e-tongue, would first of all provide a means to pace up with the ever-increasing threat of fraud. Secondly, consumer choice and purchasing options would be guaranteed.

Thus, the aim of this study was to characterize nine important physico-chemical properties (pH, color, water activity, water-holding capacity, dry matter content, and texture) of four different beef breeds and domesticated buffalo using conventional methods and sensory analysis. The potential of e-tongue to rapidly discriminate the different meat species and predict the nine physical parameters was also performed, in order to determine the feasibility of using the e-tongue both independently and as a correlative technique in combination with conventional methods.

## 2. Materials and Methods

### 2.1. Sampling and Experimental Design

Angus, domestic buffalo, Hungarian Grey, Hungarian Spotted cattle, and Holstein breed meat samples were collected from two slaughterhouses in central Hungary (Pest county) on the day of slaughtering. The animals were kept at two farms in the central part of Hungary (Pest county). The animals were of the same sex (bull) and same maturity: ready for slaughtering (their weights were nearly identical and ranged between 405 and 445 kg).

Feeding and housing were also identical (semi-intensive fattening) for all the tested animals, and they were slaughtered in the same way: resting time, stunning method, cooling technology, etc., were identical. Samples were stored uniformly at 5 °C until the experiments.

**Figure 1:**
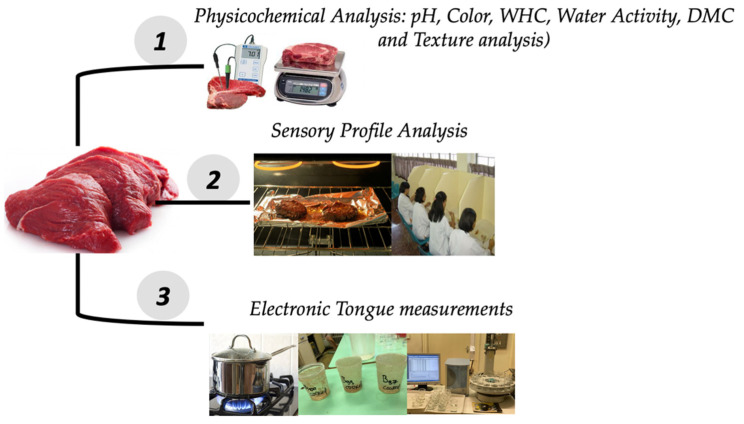
Graphical representation of the experimental design for the correlative analysis of different cattle species.

Three parallel animals were used for each of the five studied breeds, resulting in three replicate samples per breed. The left chuck steak part of the animals, which mostly contained longissimus dorsi muscles, was tested (uniformly closer to the cervical side of the cattle). The samples were each, subjected to different physico-chemical analysis, sensory profile analysis, and electronic tongue measurements (Figure 1).

### 2.2. Physico-Chemical Analysis

#### 2.2.1. Determination of pH

The pH was determined as described by Prommachart et al. [5], using a Testo 206 pH-meter (Testo GmbH, Wiena, Austria), by direct measurement in muscle, in comminuted meat using an immersion probe.

#### 2.2.2. Determination of Color

The color properties were determined as described by Aroeira et al. [27]. A hand-held tristimulus color analyzer Chromameter (CR 200, Minolta, Japan) to measure the colour after calibrating with a white standard. The sample holder of the instrument was filled with the samples with white background. The samples were acquired from the chuck steak part of the animals, which mostly contained longissimus dorsi muscles. The color was recorded through the special glass using the CIELab uniform color space at room temperature. Color of the different meat samples was then determined, according to the CIE (Commission Internationale l’Eclairage) color classification, based on three dimensions (L*: brightness; a*: red to green color; and b*: yellow to blue color). Each meat sample was measured ten times. Total color difference (ΔE), was evaluated by comparing the overall color differences of the different meat species using the Hungarian Grey as reference and the formula:

$$\Delta E*=\sqrt{\Delta {L*}^{2}+\Delta {a*}^{2}+\Delta {b*}^{2}}$$

According to the international scale, the following ranges can be applied in the total color difference (ΔE): not noticeable (0–0.5), slightly noticeable (0.5–1.5), noticeable (1.5–3.0), well visible (3.0–6.0), and great difference (6.0–12.0).

#### 2.2.3. Determination of Water-Holding Capacity (WHC)

The WHC was characterized according to Huff-Lonergan et al. [28]. An amount of sample between 200 and 300 mg was exactly weighed and put on a known weight 2500 mm^2^ (50 × 50 mm) area filter paper. The filter paper and the samples were placed between two glass plates and pressurized with 0.5 kg weight for 5 min then the filter paper was dried. The formed spot (moisture from the sample) was cut out from the filter paper and the paper without spot was weighted. The released water from sample was calculated as the proportion of area and weight of spot and sample and was expressed in [mm^2^/mg] dimension. Three replicates were analyzed.

#### 2.2.4. Determination of Water Activity

Determination of water activity was carried out by using the LabMaster-aw neo equipment (Novasina, Switzerland). A thin layer of meat was placed in the jar belonging to the apparatus, such that it covered the whole jar before the water activity values were recorded after 25 min.

#### 2.2.5. Determination of Dry Matter-Content

Moisture was determined using the AOAC method of proximate analysis [29]. Twenty grams of each sample were minced and homogenized by stirring with a laboratory stirring rod before weighing exactly 0.5 g into a pre-weighed Petri dish and placing in a hot air oven, maintained at 105 °C, for a period in which sample’s weight became constant. The samples were cooled in a desiccator to room temperature and the loss in weight was calculated as a percentage using the formula:

$$\text{Dry matter}-\mathrm{content}\left(\%\right)=\frac{\text{weight of dried sample}}{\text{weight of wet sample}}\times 100$$

#### 2.2.6. Texture Analyses

Texture was measured by Warner–Bratzler shear force (WBSF) with a SMS (Stable Micro Systems, Godalming, United Kingdom) TA.XT plus texture analyzer. Before the measurement, 150–200 g of meat from the five different species were heat-treated until the core temperature of the meat reached 72 degrees celcius (°C), then cooled to 4–6 °C after heat treatment. They were then cut into sizes of 1 × 1 × 10 cm before measuring and recording their texture values [30].

### 2.3. Sensory Evaluation

The meat samples were first vacuumed, then placed in a 72 °C water bath for 1 h. Afterwards, they were cooled on an ice bed and baked for ten minutes on a baking tray preheated to 200 °C, before commencing sensory evaluation.

Sensory quality was evaluated with the profile analysis method [31]. The panel consisted of 8 expert panelists, tests were performed in a sensory laboratory equipped with computerized booths with standardized lighting [32]. Samples were sliced and coded with three-digit random numbers. The presentation order of the samples was randomized. Panelists received water and a slice of white bread as taste neutralizer. The analyzed attributes were the followings: color hue, color vividness, softness, chewability, juiciness, fattiness, global odor intensity, cooked beef odor, global flavor intensity, cooked beef flavor, and flavor persistency. Panelists evaluated the attributes on unstructured line scales. One of the samples, Hungarian Spotted cattle was applied as a reference; this was fixed at a given point of the scales (55%), thus reducing the magnitude of the internal variation of the scale values. Data acquisition was performed with the ProfiSens sensory software developed by sensory analysis experts at the Department of Postharvest Science, Trade and Sensory Evaluation, Institute of Food Science and Technology, Hungarian University of Agriculture and Life Sciences [33].

### 2.4. E-Tongue Analysis

Forty grams of the same body part (chuck steak part) was taken from each of the three different cattles for each breed under study, resulting in three replicate samples per breed. The meat samples were separately cooked in a pot containing 600 mL of distilled water, while covered, until their core temperatures reached 72 °C.

The meat extracts obtained after cooking the three different meat samples from each breed was diluted up to 50% using distilled water to obtain a total of 100 mL solution per each sample (animal). All the diluted samples were then transferred in 100 mL beakers before putting them in the e-tongue autosampler for analysis. The volume of each tested sample during the measurement was 100 mL, the sampling time was 120 s, the sampling frequency was 1 s, and the cleaning time with distilled water was 20 s. All experiments were performed at room temperature.

The e-tongue used for the measurements was a potentiometric electronic tongue (e-tongue) with food grade sensors (BB, HA, ZZ, GA CA, JE, and JB) and was configured according to the manufacturers (AplhaMOS, Toulouse, France) recommendations prior to each adulteration measurement. To configure the instrument, a conditioning was performed using 0.01 M hydrochloric acid solution and distilled water then, a calibration using the solution prepared from the extraction of meat samples. The purpose of this, was to achieve good sensor signals from the instrument during measurement and so as to allow rapid detection of low concentration differences in the meat mixture samples otherwise, known as fingerprinting. The main operating principle of measurement for the e-tongue is based on the difference in potential changes of sensor probes (BB, HA, ZZ, GA CA, JE, and JB) against a reference electrode in zero-current conditions.

Each diluted meat extract was measured four times, resulting in 12 readings in total, from the e-tongue sensor for each beef species. The last 10 s of the sensor signals was then exported for multivariate data analysis because the signals at this point are considered to be the most stable. All data analysis was performed in R-project statistical software.

### 2.5. Statistical Data Analysis

Physico-chemical results of all the different breeds were analyzed in excel using bar charts and standard deviations. Analysis of variance (ANOVA) was used to determine significant differences between the breeds, based on their measured parameters. The Tukey method was applied for multiple comparison among breed group means considering *p* < 0.05 as significant. Sensory data were also analyzed with one-way ANOVA and Tukey’s test for least significant difference tests.

E-tongue results were analyzed by using linear discriminant analysis (LDA) for multi-class classification of the different breeds in R-project. Cross validation was performed for the LDA model to evaluate their robustness in predicting the breeds. For this, the data were divided into a training set and a validation set. The training set was made up of two-third of the data; thus, the sensor signals from the first and second replicates of each sample (body part of two animals per breed), representing six e-tongue readings. The validation set was made up of sensor signals from the third replicate (body part of one animal per breed) representing three e-tongue readings. The data splitting was performed three times, such that each sample was used at least once in the calibration and validation set.

Partial least squares (PLS) regression was used to develop models, in order to regress on some of the physico-chemical properties of the meat samples. Cross-validation was also used to test the robustness of the models by splitting the data into two groups: the calibration and validation sets. The training set consisted of two-thirds of the data, which included sensor signals from the first and second replicates of each meat breed. The validation set consisted of sensor signals from the third replicate. The data splitting was repeated as many times as possible to ensure that each replicate was used for calibration and validation at least once. The statistical parameters used to evaluate the performance of the PLS regression models were the root mean square error of calibration (RMSEC) and the coefficient of determination (R2C). For cross-validation, it was root mean square error of cross-validation (RMSECV) and coefficient of determination after cross-validation (R2CV). The optimum number of latent variables was determined based on the minimum RMSECV value.

## 3. Results

### 3.1. Physico-Chemical Analysis

#### 3.1.1. pH

**Figure 2:**
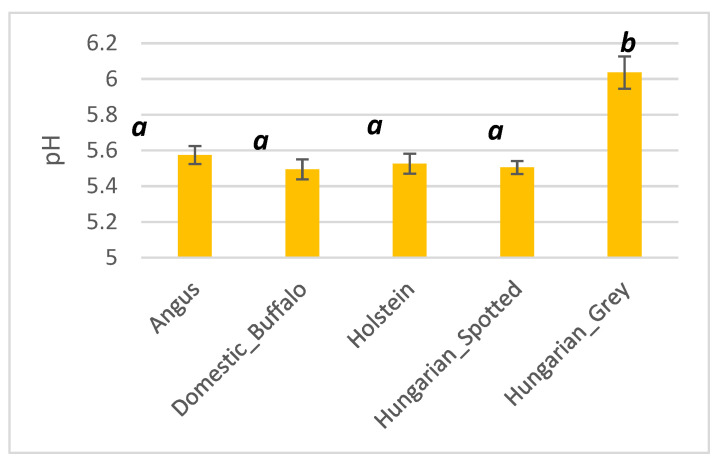
Bar plot showing the pH of Angus, domestic buffalo, Holstein, Hungarian Spotted, and Hungarian Grey bovine breeds. Different superscripts imply significant differences.

With an average pH of 6.04, the Hungarian Grey species had the highest pH among all the other species, whilst the domestic buffalo had the lowest with an average pH of 5.49 (Figure 2). Angus, Holstein, and the Hungarian Spotted cattle had average pH of 5.57, 5.53, and 5.50, respectively. There were significant differences (*p* < 0.05) between the pH of the Hungarian Grey species and Angus, domesticated buffalo, Holstein, and Hungarian Spotted cattle.

#### 3.1.2. Color

**Figure 3:**
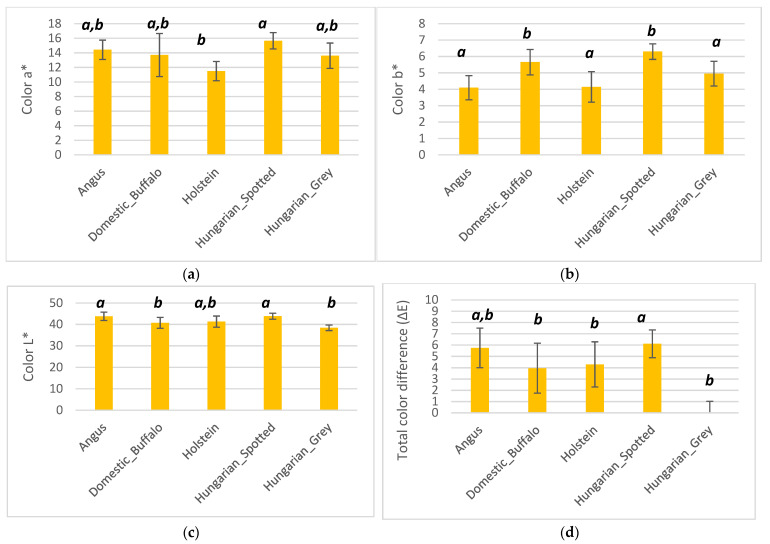
Bar plot showing the color a* (**a**), color b* (**b**), color L* (**c**) and total color difference (**d**) values of Angus, domestic buffalo, Holstein, Hungarian Spotted, and Hungarian Grey bovine breeds. Different superscripts imply significant differences.

Color parameters: a* (a), b* (b), L* (c), and total color difference (d) measurements of the five different breeds are presented in Figure 3. The Hungarian Spotted breed had the highest average color a* (15.66), color b* (6.29), and color L* (43.84) values. Holstein had the lowest average color a* (11.49), whereas Angus had the lowest average color b* (4.09) and the Hungarian Grey had the lowest color L* (39.51) (Figure 3a). There were significant differences (*p* < 0.05) between the redness (color a* values) of Angus and Holstein, and also between Holstein, Hungarian Grey, and Hungarian Spotted cattle. Hungarian Grey and Hungarian Spotted cattle were also significantly different in terms of redness. Holstein and Angus were also significantly different in terms of color a*. Yellowness (Color b* values) of Hungarian Spotted breed, Holstein, Hungarian Grey, and Angus were significantly different (*p* < 0.05). There were also significant differences between the color b* values of the domestic buffalo, Holstein, and Angus. For color L*, which represents lightness and brightness, significant differences (*p* < 0.05) were observed between the Angus and domestic buffalo, as well as between the Angus and Hungarian Grey breeds. Hungarian Spotted cattle were also significantly different from the Hungarian Grey. The Hungarian Grey cattle breed was used as the reference for the determination of total color difference because it had the highest pH. Based on the calculated average of the total color differences, there was a clearly visible difference (3.0–6.0) between Angus, domestic buffalo, and Angus species. There was also considerable difference between the Hungarian Spotted cattle and the other breeds as proven in the Anova analysis where total color difference was significant (*p* < 0.05) between Angus, domestic buffalo, and Hungarian Spotted cattle meat. There was also significant different the Hungarian Spotted cattle, Holstein, and Hungarian Grey. There was no significant difference between the domestic buffalo and Hungarian Spotted cattle meat in (Figure 3d).

#### 3.1.3. Water-Holding Capacity

**Figure 4:**
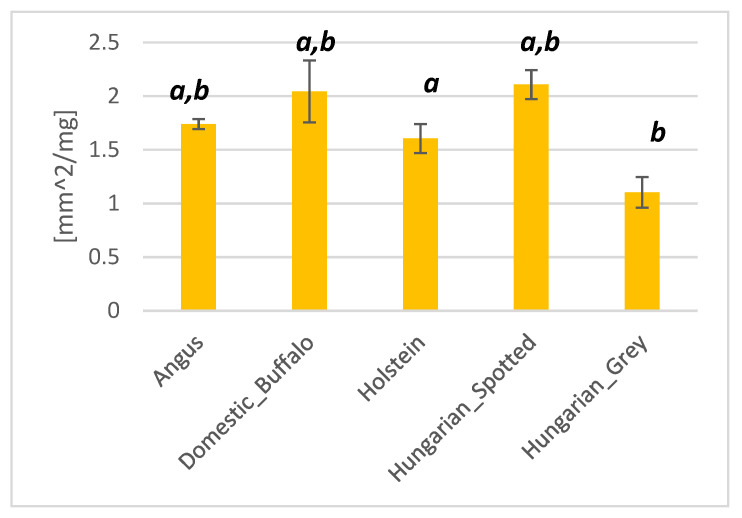
Bar plot showing the water-holding capacity of Angus, domestic buffalo, Holstein, Hungarian Spotted, and Hungarian Grey bovine breeds. Different superscripts imply significant differences.

The Hungarian Spotted breed had the highest average water-holding capacity of 2.11 mm^2^/mg, compared to the lowest water-holding capacity of 1.10 mm^2^/mg recorded for the Hungarian Grey breed (Figure 4). Angus, domestic buffalo, and Holstein had an average water-holding capacity of 1.74, 2.04, and 1.60, respectively. There were significant differences (*p* < 0.05) between the water-holding capacity of the Hungarian Grey breed and all the other breeds. The water-holding capacity of the Hungarian Spotted breed and Holstein were also significantly different.

#### 3.1.4. Water Activity

**Figure 5:**
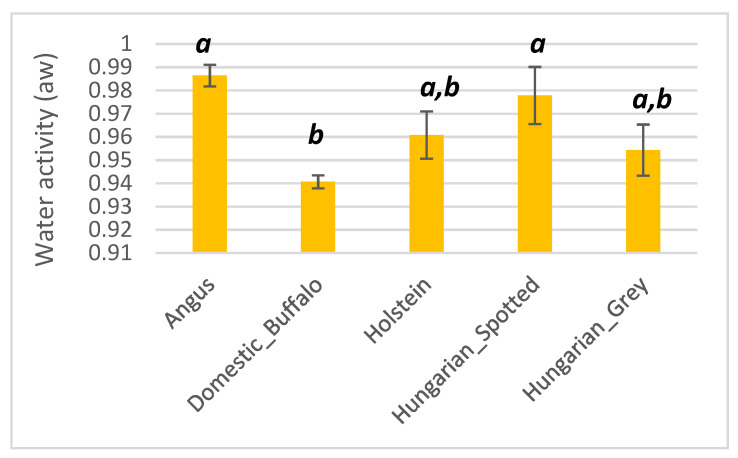
Bar plot showing the water activity of Angus, domestic buffalo, Holstein, Hungarian Spotted, and Hungarian Grey bovine breeds. Different superscripts imply significant differences.

Angus had the highest average water activity of 0.99, whilst the domestic buffalo had the lowest (0.94) (Figure 5). Holstein, Hungarian Spotted cattle, and Hungarian Grey had average water activity of 0.96, 0.97, and 0.98, respectively. There was a significant difference (*p* < 0.05) between the water activity of Angus and domestic buffalo cattle meat and also, between the domestic buffalo and Hungarian spotted cattle breeds, as shown in Figure 5.

#### 3.1.5. Dry Matter Content

**Figure 6:**
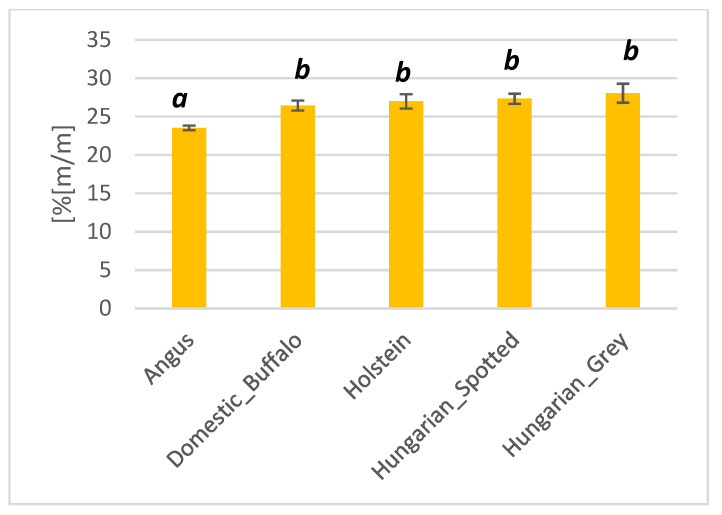
Bar plot showing the dry matter content of Angus, domestic buffalo, Holstein, Hungarian Spotted, and Hungarian Grey bovine breeds. Different superscripts imply significant differences.

All the five different breeds had dry matter content higher than 23% *m*/*m* (Figure 6). The Hungarian Grey breed had the highest average dry matter content of 28.04% *m*/*m*, whilst Angus had the lowest dry matter content of 23.51% *m*/*m*. The domestic buffalo, Holstein and Hungarian Spotted breed had average dry contents of 26.43, 26.98, and 27.32% *m*/*m*, respectively. Significant differences (*p* < 0.05) were only observed between the dry matter content of Angus and all the other breeds.

#### 3.1.6. Warner–Bratzler Shear Force

**Figure 7:**
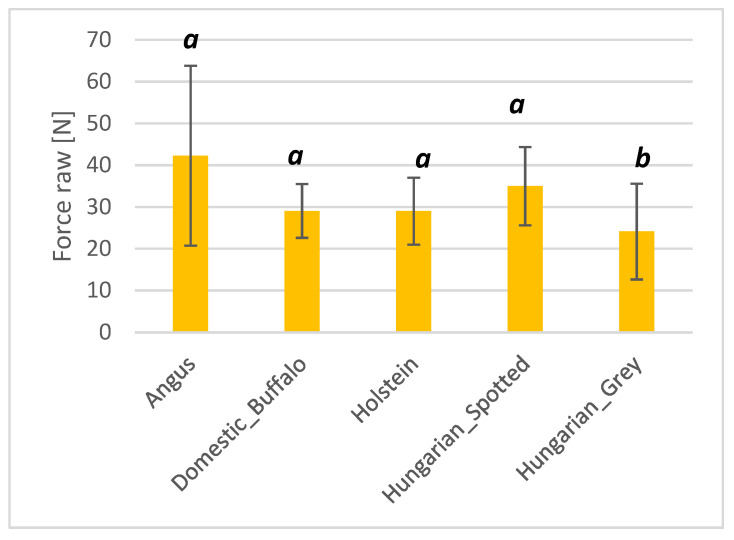
Bar plot showing the measurement of force values of Angus, domestic buffalo, Holstein, Hungarian Spotted, and Hungarian Grey bovine breeds. Different superscripts imply significant differences.

The Warner–Bratzler shear force was only significantly different between the Hungarian Grey cattle and the other breeds, as shown in Figure 7. The high standard deviations may be attributed to the inhomogeneity of the meat muscles and fibrous constituents.

### 3.2. Sensory Evaluation

**Figure 8:**
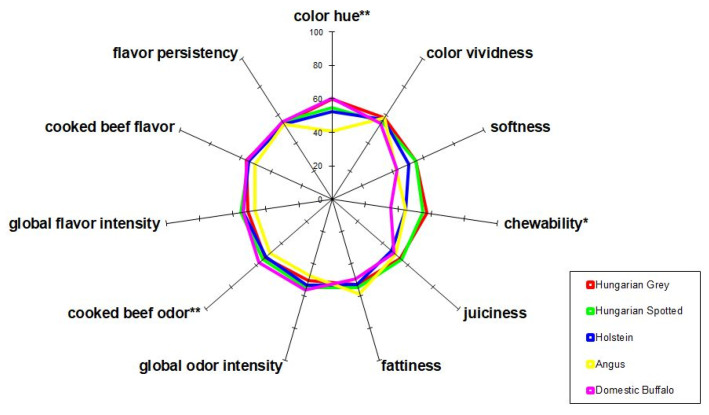
Sensory profile of the meat samples. Key: *; significant, **; very significant.

Based on the sensory perception (Figure 8) of the color, Angus had significantly lighter color (*p* < 0.01), while in the vividness of the color the panel detected no difference. Among the texture attributes the chewability of the domestic Buffalo had the lowest scores, both the Hungarian Grey cattle and the Hungarian Spotted cattle were easier to chew (*p* < 0.05). The cooked beef odor was the most intense in case of the domesticated buffalo, followed by the Hungarian Spotted cattle and the Hungarian Grey cattle. In the remaining attributes there were no significant differences.

### 3.3. Classification of Meat Breeds with E-Tongue

**Figure 9:**
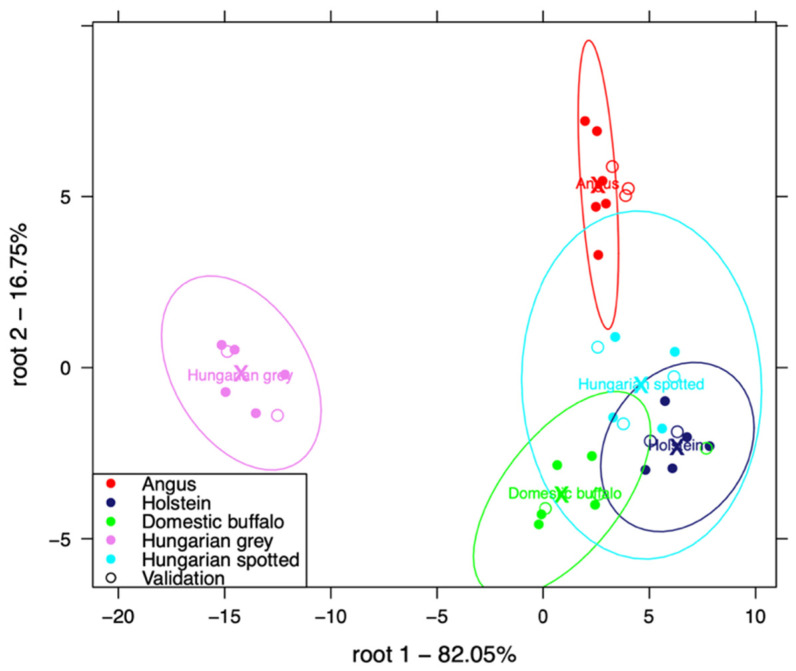
Classification plot of Angus, domestic buffalo, Holstein, Hungarian Spotted, and Hungarian Grey bovine breeds with e-tongue.

Figure 9 shows the classification of the five different meat breeds with e-tongue using three-fold, cross-validation in a linear discriminant analysis (LDA). The Hungarian Grey breed and Angus could be clearly separated in the plot, but the confidence ellipse of Holstein, domestic buffalo, and the Hungarian Spotted breed appeared to overlap.

The confusion matrix (Table 1) for this classification showed that all the different breeds could be classified with 100% average recognition accuracy (calibration accuracy) and 97.52% average prediction accuracy (accuracy with cross-validation). All the different breeds could be classified with 100% accuracy with cross-validation, except for 12.41% of the domestic buffalo, which were misclassified as the Hungarian Spotted breed. Table 2 also, shows the PLSR prediction of physico-chemical parameters in domestic buffalo, Holstein, Hungarian Spotted, and Hungarian Grey bovine breeds, where pH was predicted with the best accuracy.

## 4. Discussion

In our study, only the Hungarian Grey cattle breed had a significantly higher pH; the pH range of all the different breeds were in agreement with those generally reported in the literature for carcasses from bovine species [7,34]. According to McWilliams [35], the pH profile of meat directly impacts meat color early postmortem because meat color is highly dependent on the stability of myoglobin molecules and enzymes involved in color development. The meat samples from Angus and the Hungarian Spotted breed were lighter (Color L*) and redder (Color a*), compared to all the cattle breeds. In terms of total color difference, these two breeds were, again, the most distinct from the rest. This was also in agreement with the results of sensory evaluation, where Angus had significantly lighter color, compared to all the other breeds. Meat from both Angus [27] and Hungarian Spotted cattle breed have been associated with lighter colors, compared to meat from the Hungarian Grey breed, which have been generally reported to be darker and not particularly marbled, since most of the suet forms subcutaneous and intestinal fat deposits [36]. In fresh meat, color is the most important attribute that consumers use as purchase criterion [17].

Water-holding capacity (WHC) is also a meat quality parameter generally related to the concept of isoelectric point (pl); as the pH of meat decreases due to the buildup of lactic acid, the pH approaches the isoelectric point, which is basically the point of pH where positive charges are equal to negative charges [28]. When positive and negative charges are equal, we find the water-holding capacity to be at its lowest level [37]; this is probably why domestic buffalo and Hungarian Spotted had the lowest pH but higher WHC’s, compared to the other breeds. Although there has not much study on the WHC of the Hungarian Spotted cattle in literature, some studies have suggested that the WHC of the domestic buffalo is comparable to some species of beef cattle as confirmed in this study. Domestic buffalos are mostly phenotypically homogenous throughout their distribution. There are no recognized breeds and they are used for draught and meat [38]. Poor WHC can lead to high drip and purge loss from meat and meat products, which can often imply a loss of weight from carcasses and cuts that may affect the yield and quality of processed meats. Improved WHC, on the other hand, leads to better protein functionality, as well as greater processing and cooking yields [39]. Therefore, selection of proteins with an appropriate WHC is vital in food formulation although, various intrinsic, extrinsic, and environmental factors, such as ionic strength, temperature and pH can affect the WHC of protein.

The influence of pH and water activity (aw) on microbial growth is an area of interest in food quality that has been widely studied. Generally, low water activity and high pH in food is recommended for microbial control. The pH and water activity were inversely related to each other in this study. For example, Angus and Hungarian Spotted breed had the highest water activity, although they had the lowest pH among all different meat breeds in this study. Hungarian Grey breed also had the lowest water activity, although it had the highest pH. Watanabe et al. [39] reported that the correlation between pH and WHC was higher than the correlation between intramuscular fat (IMF) and WHC in five Japanese pork breeds. They concluded that pH was one of the most important factors influencing WHC in pork meat even though fat content fat content affects WHC because it is nonpolar (no water-holding) and it decreases the proteins available for attracting and holding water.

Dry matter content, which can refer to the material remaining after the removal of water, was highest in the Hungarian Grey breed. The dry matter content of meat has been often related to the Warner–Bratzler shear force of meat, which is a criterion of measurement for meat tenderness. The Warner–Bratzler shear force results were in agreement with chewability results in the sensory analysis, where meat from the Hungarian Grey breed was the only one that was significantly different from the other breeds. Hungarian Grey is one of the most famous Hungarian breeds, a breed native to Hungary; parts of the body that are valuable for meat production are immature, i.e., the rump is poor in muscle, the thighs are thin, and the flesh is dry and less fatty. Dry matter content was only significantly lower in the Angus breed, which is known for traits that can make a real difference in cow herd profitability, including calving ease, marbling, growth, and superior milking capabilities, particularly with high fertility rates; however, they are not usually reared for their meat [3].

Meat quality, as perceived by consumers, is a subjective, multidimensional, and dynamic concept. For this reason, other analytical instruments, such as the electronic tongue, have been recommended for objective validation of meat quality. Classification models developed with the electronic tongue showed that the instrument could classify Angus, Holstein, Hungarian Spotted, and Hungarian Grey breeds with 100% accuracy. Only domestic buffalo meat showed misclassifications as meat from the Hungarian Spotted breed. This suggests that, although meat quality parameters of the domestic buffalo have been reported in literature [40] and also proven through physico-chemical and sensory evaluations in this study to be similar to beef breeds, the electronic tongue was still sensitive enough to show that it is actually not a beef breed. Cattle meat classification is often performed through genotyping technologies that use methods such as the diallelic and microsatellite marker techniques. Some studies have suggested that developments in automated genotyping methods may ultimately favor the use of diallelic markers, because these technologies will allow many hundreds of loci to be scanned, but this is also time-consuming and expensive, compared to electronic tongue measurements [41]. Additionally, microsatellite markers have been reported to be more powerful than diallelic markers, but genotyping error can be high and it is difficult to compare genotypes typed in different laboratories [41]. According to Bressan et al. [42], meats originating from different finishing systems could be reliably discriminated, essentially on the basis of their fatty acid profile, but global distinction of meat from the two genetic groups was not reliable. Other methods, such as the multiplex mPCR analysis of DNA and DART–HRMS analysis of the TAGs profile of beef, have been effectively used to discriminate cattle breedx [43]. The authors, however, concluded that the DART–HRMS method is primarily a screening method for testing a large number of samples and selecting only suspect specimens, which are then confirmed by DNA analysis, so it may not be cost-effective. They also concluded that PCRs analysis can be used for precise animal species identification; however, it takes more time to get results.

The electronic tongue on the other hands, is a quick and reliable method with a very short analysis time. As a correlative technique in our current study, the electronic tongue could also predict, pH, water activity, and dry matter content of all the different meat breeds with acceptable accuracies. The pH could be predicted with the best accuracies in all the different meat breeds. This is particularly important because, as reported in the literature and proven in our results, a low ultimate pH results in meat proteins having decreased water-holding capacity and a lighter color. Conversely, a higher ultimate pH will give a darker color and less drip loss, which also affects eating quality characteristics, such as juiciness, tenderness, and taste [44].

## 5. Conclusions

Modeling and determination of meat origin and physico-chemical properties is a promising step that can help monitor increasing cases of meat fraud (misrepresentation) and also help consumers make the right decision, with regards to meat preference. In our study, the purported meat quality differences between five prominent cattle breeds were ascertained using both conventional and advanced rapid methods. Our results suggest that, although physico-chemical analysis and sensory evaluation have both proven to be effective methods of meat characterization, the electronic tongue presents higher sensitivities of component detection that be used to rapidly discriminate meat quality in the studied different beef breeds. This especially important as meat origin and quality have the potential to impact both economic revenue in the meat industry, as well as consumer perception. It is also important to note that our study only proves the applicability and effective of this corelative technique. Further studies would be required if they are to be applied to discriminate other breeds. Further study is, however, recommended to strengthen the robustness of LDA and PLSR prediction models.

## Figures and Tables

**Table 1:** Confusion matrix showing the classification accuracies of Angus, domestic buffalo, Holstein, Hungarian Spotted, and Hungarian Grey bovine breeds. Columns represent the actual class membership (%) and the rows represent the predicted class membership (%).

		Angus	Holstein	Domestic Buffalo	Hungarian Grey	Hungarian Spotted
Average recognition: 100%	Angus	100	0	0	0	0
	Holstein	0	100	0	0	0
	Domestic buffalo	0	0	100	0	0
	Hungarian Grey	0	0	0	100	0
	Hungarian Spotted	0	0	0	0	100
		**Angus**	**Holstein**	**Domestic Buffalo**	**Hungarian Grey**	**Hungarian Spotted**
Average prediction: 97.52%	Angus	100	0	0	0	0
	Holstein	0	100	0	0	0
	Domestic buffalo	0	0	87.59	0	0
	Hungarian Grey	0	0	0	100	0
	Hungarian Spotted	0	0	12.41	0	100

**Table 2:** PLSR prediction of physico-chemical parameters in domestic buffalo, Holstein, Hungarian Spotted, and Hungarian Grey bovine breeds.

Parameter	R^2^C	RMSEC	R^2^CV	RMSECV
pH	0.89	0.07	0.73	0.10
Water activity	0.78	0.01	0.58	0.01
Dry matter content [%(m/m)]	0.78	0.01	0.58	0.01
Water-holding capacity [mm^2^/mg]	**	**	**	**
Color a*	**	**	**	**
Color b*	**	**	**	**
Color L*	**	**	**	**
Force raw [g]	**	**	**	**
Force roasted [g]	**	**	**	**

**: Could not be predicted.

## Data Availability

The data presented in this study are available on request from the corresponding author. The data are not publicly available, due to privacy and ethical reasons.
